# The anatomical placode in reptile scale morphogenesis indicates shared ancestry among skin appendages in amniotes

**DOI:** 10.1126/sciadv.1600708

**Published:** 2016-06-24

**Authors:** Nicolas Di-Poï, Michel C. Milinkovitch

**Affiliations:** 1 Laboratory of Artificial and Natural Evolution, Department of Genetics and Evolution, University of Geneva, 1211 Geneva, Switzerland.; 2 Research Program in Developmental Biology, Institute of Biotechnology, University of Helsinki, 00014 Helsinki, Finland.; 3 SIB Swiss Institute of Bioinformatics, 1211 Geneva, Switzerland.

**Keywords:** Skin appendages, scales, reptiles, snakes, lizards, crocodiles, anatomical placode, signalling placode, EDA, evo-devo

## Abstract

Most mammals, birds, and reptiles are readily recognized by their hairs, feathers, and scales, respectively. However, the lack of fossil intermediate forms between scales and hairs and substantial differences in their morphogenesis and protein composition have fueled the controversy pertaining to their potential common ancestry for decades. Central to this debate is the apparent lack of an “anatomical placode” (that is, a local epidermal thickening characteristic of feathers’ and hairs’ early morphogenesis) in reptile scale development. Hence, scenarios have been proposed for the independent development of the anatomical placode in birds and mammals and parallel co-option of similar signaling pathways for their morphogenesis. Using histological and molecular techniques on developmental series of crocodiles and snakes, as well as of unique wild-type and EDA (ectodysplasin A)–deficient scaleless mutant lizards, we show for the first time that reptiles, including crocodiles and squamates, develop all the characteristics of an anatomical placode: columnar cells with reduced proliferation rate, as well as canonical spatial expression of placode and underlying dermal molecular markers. These results reveal a new evolutionary scenario where hairs, feathers, and scales of extant species are homologous structures inherited, with modification, from their shared reptilian ancestor’s skin appendages already characterized by an anatomical placode and associated signaling molecules.

## Teaser

Researchers show that scales, feathers, and hairs of reptiles, birds, and mammals evolved from the scales of their common reptilian ancestor.

## INTRODUCTION

Extant amniotes exhibit lineage-specific skin appendages: hairs in mammals, feathers (and feet scales) in birds, and various types of scales in reptiles. With the exception of face and jaw scales in crocodilians, which form through a process analogous to material cracking (*1*), the development of all reptilian scales is preceded by the patterning of the skin into discrete developmental units through reaction-diffusion (*2*), a mechanism also observed for the development of mammalian hair and bird feathers. However, whether this very general process suffices to demonstrate the homology among amniote skin appendages has been debated for years (*3*–*9*). Hairs, feathers, and scales exhibit substantial developmental specificities, blurring evolutionary relationships among the processes involved. One primary example of developmental divergence among skin appendage types is that hairs, feathers, and avian and turtle scutate scales develop from a characteristic local thickening of the epidermis [the anatomical placode (*10*–*14*)], whereas all authors agree that scales in squamates (snakes and lizards) form from regular dermoepidermal elevations without exhibiting placodes (*3*, *9*, *14*–*17*). Later developmental stages are even more divergent as hair and feather placodes are associated with a dermal condensation and further develop into follicular organs characterized by substantial downward growth (hair follicle) or outgrowth (feather follicle) of the epidermis, whereas mature scales typically develop by asymmetrization of the initial dermoepidermal elevations without showing any apparent sign of dermal condensation.

Several studies (*9*, *18*–*23*) have shown that conserved signaling pathways, evidenced by the expression of the Sonic hedgehog (*Shh*), β-catenin (*Ctnnb1*), ectodysplasin A receptor (*Edar*), and/or bone morphogenetic protein (*Bmp*) genes, are involved in skin patterning and early morphogenesis of all amniote skin appendages, including avian and crocodilian scales, turtle scutes, mammalian hairs, mammary glands, and avian feathers. This led to the recent proposition (*9*) that placodes should be defined as localized molecular signaling centers (hence, these should be considered homologous in all amniote skin appendages) that can form without the presence of an “anatomical placode.” Conversely, other authors (*3*, *5*, *8*) argue that skin appendages have evolved independently in reptiles, birds, and mammals and that similarities in signaling are due to independent co-option of these molecular pathways.

Here, we show for the first time that the development of scales in different reptilian lineages, including squamates, is actually associated with the presence of an anatomical placode presenting all the characteristics observed in avian and mammalian placodes: (i) an epidermal thickening with columnar cells exhibiting reduced proliferation rate; (ii) typical spatial expression of placode molecular markers such as *Shh*, *Ctnnb1*, and *Edar*; and (iii) localized and conserved signaling in the dermis underlying the placode, such as *Bmp4*, readily suggesting an evolutionary developmental link with the dermal condensate observed in birds and reptiles. We show that anatomical placodes in reptiles have been overlooked in previous studies, most likely because they form very transitorily in time and nonconcurrently in space; that is, they are difficult to identify on any specific embryo because they establish multiple tracts whose developmental timing and locations vary across the body.

These results are additionally supported by our analysis of a scaleless phenotype in the bearded dragon (*Pogona vitticeps*), a codominant mutation that we identify as an in-frame deletion of 14 amino acids in a highly conserved tumor necrosis factor (TNF) motif of the EDA protein. Comparing skin morphogenesis and signaling in wild-type and scaleless dragons, we demonstrate that the latter fail in the development of placodes, both as anatomical entities and as signaling centers, confirming the requirement of an anatomical placode for proper morphogenesis of all skin appendages in amniotes.

This set of new results coherently and conclusively indicates that most skin appendages in amniotes are homologous; that is, they all evolved from a shared common ancestor that exhibited appendages developing from an anatomical placode and expressing a set of signaling molecules still involved in the development of scales, hairs, and feathers of extant species.

## RESULTS

### Reptilian scales develop from an anatomical placode

**Fig. 1:**
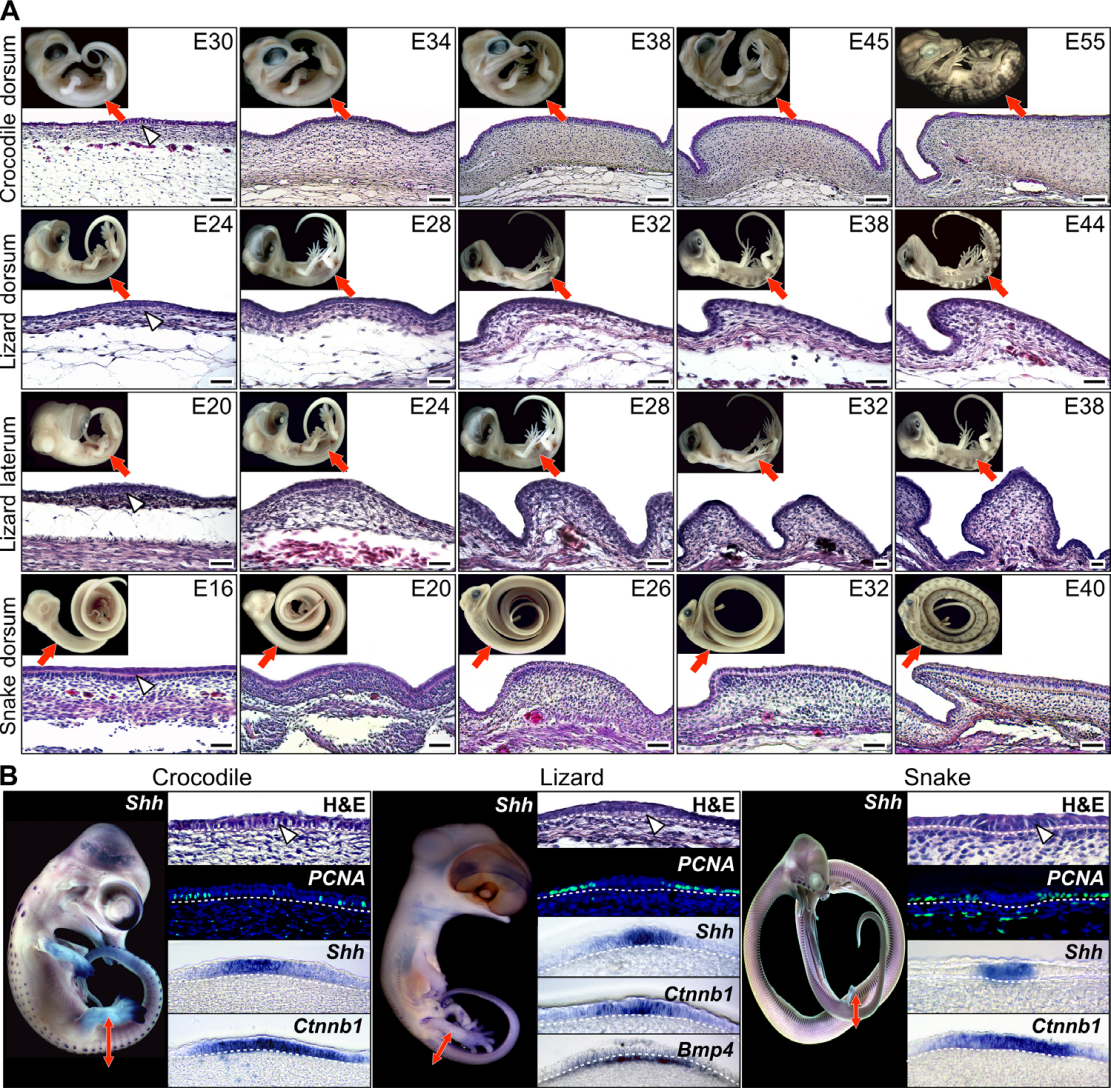
Development of epidermal scales during reptilian embryogenesis. (**A**) Hematoxylin and eosin (H&E) staining of skin sections from different body regions (indicated with red arrows on the top insets with lateral views of corresponding embryos) of *C. niloticus* (crocodile; top row), *P.vitticeps* (lizard; two middle rows), and *P. guttatus* (snake; bottom row) embryos at various developmental stages [indicated as embryonic days (E) after oviposition]. White arrowheads indicate the anatomical placode. Scale bars, 100 μm. (**B**) Anatomical placodes in *C. niloticus* (left panels), *P.vitticeps* (middle panels), and *P. guttatus* (right panels) embryos. For each species, the whole-embryo WMISH with Sonic hedgehog (*Shh*) is shown (left panel) as well as, from top to bottom, high magnification of H&E-stained placode sections (white arrowheads indicate placode columnar cells), immunohistochemistry with PCNA (proliferation marker; epidermal-dermal junction indicated by dashed white lines), and parasagittal cryosections of placodes after *Shh* or β-catenin (*Ctnnb1*) WMISH. *Bmp4* is also shown for lizard. Red double-headed arrows indicate the body region processed for sectioning.

Our serial sectioning and histological analyses of skin developmental series (Fig. 1A) in crocodiles (*Crocodylus niloticus*), bearded dragon lizards (*P. vitticeps*), and corn snakes (*Pantherophis guttatus*) confirm the results of previous studies (*1*, *4*, *6*, *24*) that indicate that early scale morphogenesis in reptiles consists of regular dermoepidermal elevations that typically further develop into oriented asymmetrical scales with various levels of overlap, depending on the species and body area. In addition, we show for the first time that each of these dermoepidermal elevations that generate scales in crocodiles, lizards, and snakes occurs at the location of a transient developmental unit that exhibits the characteristics (Fig. 1B) of the mammalian and avian anatomical placode. First, the epidermis shows distinctive columnar upright cells that generate the characteristic epidermal thickening also observed in hair and feather placodes (*10*, *11*). Second, our proliferating cell nuclear antigen (PCNA) analyses indicate a reduced proliferation rate of the placode epidermal cells as observed in mouse and chicken (*25*, *26*). Third, using whole-mount in situ hybridization (WMISH) with species-specific probes, we show that crocodile, lizard, and snake placodes all exhibit spatial expression of *Shh* in a nested subpopulation of the *Ctnnb1*-expressing epidermal cells, as previously observed in mammalian hair and bird feather placodes (*27*, *28*). Fourth, using in situ hybridization, we show *BMP4* signaling in the dermis underlying the lizard scale placode (Fig. 1B). Although we could not unambiguously confirm it in snakes, this result in lizards suggests that dermal BMP signaling under the placode is an ancestral characteristic for all amniotes and that it preceded the development of a dermal condensate in birds and reptiles during evolution.

### Multiple scale tracts generate macropatterning of reptilian scales

**Fig. 2:**
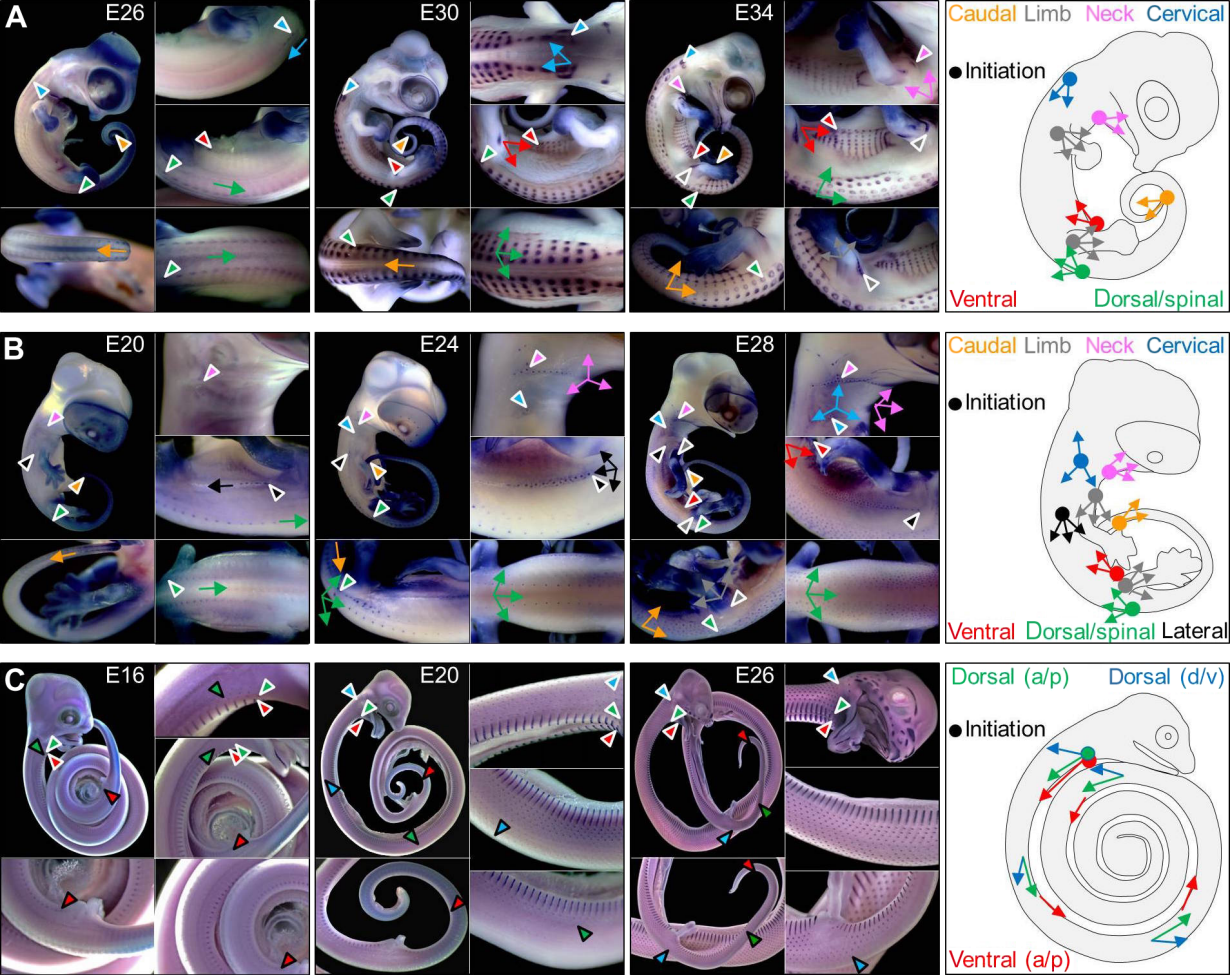
Macropatterning of developing scales in reptiles. (**A** and **B**) WMISH with *Ctnnb1* in *C. niloticus* and *P. vitticeps* embryos at various developmental stages. Arrowheads indicate the initiation sites of scale tracts and arrows indicate the directions of scale tracts. Colors correspond to different tracts schematically represented in the right panels (dots, initiation sites; arrows, directions of development). (**C**) WMISH with *Shh* in *P. guttatus* embryos at various developmental stages. Arrowheads with white borders indicate tract initiation sites, and arrowheads with black borders indicate the boundaries of *Shh* expression at different developmental stages, showing the different anteroposterior (a/p) and ventrodorsal (v/d) gradients (see schematic in the right panel).

In chicken, feathers are organized into discrete tracts associated to different body areas (*29*). This macropatterning is particularly visible, even at the adult stage, by the presence of bare skin between the tracts. Our WMISH experiments, with early developmental scale markers, such as *Shh* and *Ctnnb1*, on developmental series of Nile crocodiles and bearded dragon lizards clearly indicate (Fig. 2, A and B) that scales over the body initiate with a similar anatomical placode [except for crocodilian facial and jaw scales (*1*)] and that macropatterning of scales involves multiple tracts whose spatiotemporal development is highly similar between the two species. Several of these tracts (caudal, spinal, cervical, ventral, humeral, and femoral) could be argued homologous to those characterized in chicken (*29*–*32*).

Despite these similarities, lineage-specific scale tracts also exist as illustrated by the presence and absence of a lateral tract that corresponds to lateral spines in bearded dragon lizards (Fig. 2B) and in Nile crocodiles (Fig. 2A), respectively (see below). The most marked and derived macropatterning of skin in reptiles is observed in snakes (Fig. 2C). We show that this lineage exhibits a highly simplified spatiotemporal developmental dynamic that involves only two tracts of developing scales: a ventral tract that shows an anteroposterior sequence of development and a laterodorsal tract that exhibits a superposed anteroposterior and ventrodorsal progression. The development of reptilian scales in a specific sequence within each tract adds to the difficulty of capturing the transient anatomical placode stage; proper observation of placodes requires sampling the skin along the ordered developmental series of a single tract.

### EDA-deficient scaleless lizards do not develop anatomical placodes

**Fig. 3:**
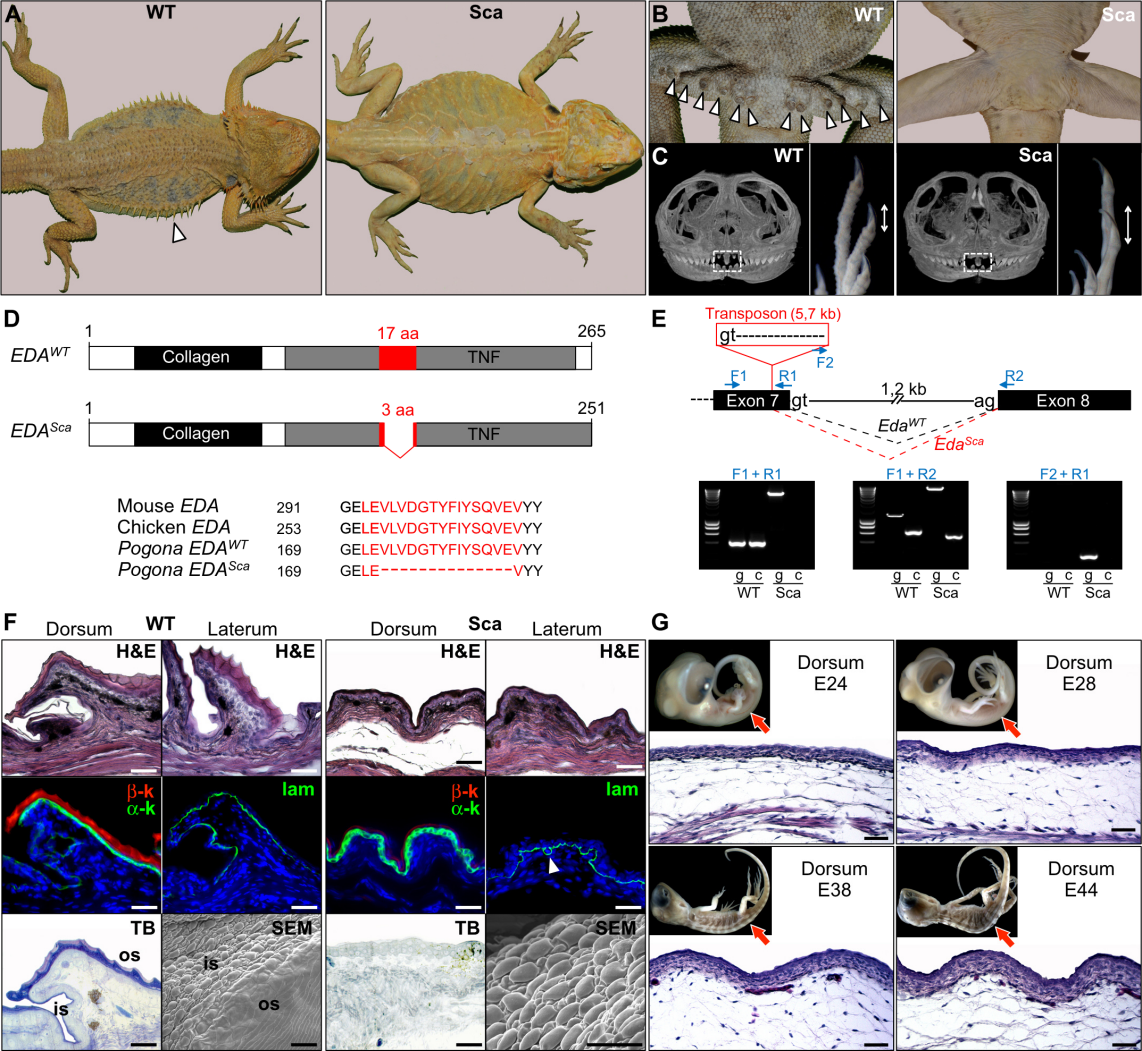
Characterization of mutant scaleless *P. vitticeps* lizards. (**A**) Dorsal views of adult wild-type (WT) and scaleless (*Sca*) *P. vitticeps* lizards. The white arrowhead indicates the presence of large lateral spines in the WT. (**B**) Ventral views of WT and *Sca* adult males showing the absence of femoral pores (arrowheads) in mutant lizards. (**C**) Micro x-ray computed tomography scan virtual sections of the skull (left) and magnified views of the autopod (right) of WT and Sca dragons at birth. White frames indicate the position of the pleurodont regenerating teeth, and double-headed arrows show the relative sizes of claws. (**D**) Diagram of WT (*EDA*^*WT*^) and mutant scaleless (*EDA*^*Sca*^) active *EDA* proteins. The conserved collagen and TNF domains are shown as black and gray boxes, respectively. The most conserved TNF motif [17 amino acids (aa) in WT] is shown in red. The mutant *EDA* protein has an in-frame deletion of 14 amino acids, as shown by the alignment of *EDA* protein sequences from mouse, chicken, and WT and Sca *P. vitticeps* (lower panel). Black numbers represent amino acid position. (**E**) Upper panel: diagram showing the genomic structure (from exon 7 to 8) of the *P. vitticeps Eda* gene. Intron length and splice donor (gt) and acceptor (ag) sites are indicated. Blue arrows show the positions of primers used for reverse transcription polymerase chain reaction (RT-PCR) analyses. In the scaleless mutant genome, a transposon of 5.7 kb starting with an alternative splice donor site is inserted in the 3′ end of exon 7, thus leading to an alternative splicing (red dashed lines) of the mutant *Eda*^*Sca*^ gene in comparison to the splicing of the *Eda*^*WT*^ gene (black dashed lines). Lower panels: RT-PCR analysis (g, on genomic DNA; c, on skin cDNA) of WT and Sca animals using the indicated primer combinations. (**F**) Top row: H&E staining of skin sections from dorsal and lateral body regions of adult WT and scaleless dragons. Middle row: immunofluorescent staining of α-keratins (α-k) and β-keratins (β-k) or laminin (lam; arrowhead shows convoluted basal membrane) in dorsal skin of adult WT and scaleless animals. Bottom row: Toluidine blue (TB) staining of dorsal skin sections and scanning electron microscopy (SEM) images of skin molts from adult WT and scaleless lizards. is, interscale region; os, outer scale region. Scale bars, 50 μm. (**G**) H&E staining of dorsal skin sections of scaleless *P. vitticeps* embryos at various developmental stages (indicated as embryonic days after oviposition); red arrows in the top insets indicate the locations of skin sections on lateral views of the corresponding embryos. Scale bars, 100 μm.

Using breeding experiments, we confirm that scaleless bearded dragons (Fig. 3A), which are available in the pet trade, are homozygous for a codominant mutation. Homozygous scaleless mutants (*Sca*/*Sca*) lack all scales on the body (ventral/dorsal scales and lateral spines; Fig. 3A) and femoral glands (Fig. 3B), and exhibit reduced dentition and (paradoxically) longer claws at birth (Fig. 3C). Such an ectodermal dysplasia syndrome is reminiscent of similar phenotypes in other vertebrates because of impairments of the EDA receptor (EDAR; a member of the TNF family) (*18*) or its ligand EDA, indicating a conserved role of this pathway in reptiles as well. Reduced expression or structural mutations of members of the EDA/EDAR pathway generate absence or abnormal development of hairs, sweat glands, mammary glands, nails, teeth, and dermal bones in mammals (*32*–*35*), and of scales, fins, plates, spines, teeth, and facial bones in fish (*36*, *37*).

We therefore used complementary DNA (cDNA) prepared from skin samples of homozygous wild-type and scaleless bearded dragons to amplify and sequence both their EDAR and EDA transcripts. Our analyses indicate that the scaleless mutation in bearded dragons is caused by an in-frame deletion of 14 amino acids within the most highly conserved TNF motif of the EDA protein (Fig. 3D) (*38*). To uncover the origin of this deletion, we amplified the 3′ end of exon 7 and its 3′ adjacent intron from genomic DNA in wild-type and scaleless individuals. Sequencing results indicate that the *Sca* allele contains a 5′ 688–base pair (bp) insertion (Fig. 3E), most of which is recognized as a transposon of the LTR-Gypsy family, which generates a new splice donor site (gt) 42 bases upstream of the wild-type donor site, thus generating a 14–amino acid deletion in the corresponding transcript (Fig. 3D).

H&E, immunohistological, and TB staining analyses indicate that scaleless dragons maintain the α-keratin layer but virtually lack both the β-layer of the epidermis and the uppermost layer of the dermis (superficial loose dermis; Fig. 3F). This indicates that the entire skin of scaleless dragon is similar in structure and composition to the narrow hinge regions (that is, the skin in between scales) of wild-type scales (Fig. 3F). In addition, laminin immunostaining shows that the epidermis basal membrane is abnormally circumvolved in *Sca*/*Sca* individuals. Scaleless dragons show an irregular skin surface with the initiation of some dermoepidermal undulations of the skin (Fig. 3G), indicating that this phenomenon does not fully require the presence of anatomical placodes.

### The *sca* mutation precludes placode proper signaling

**Fig. 4:**
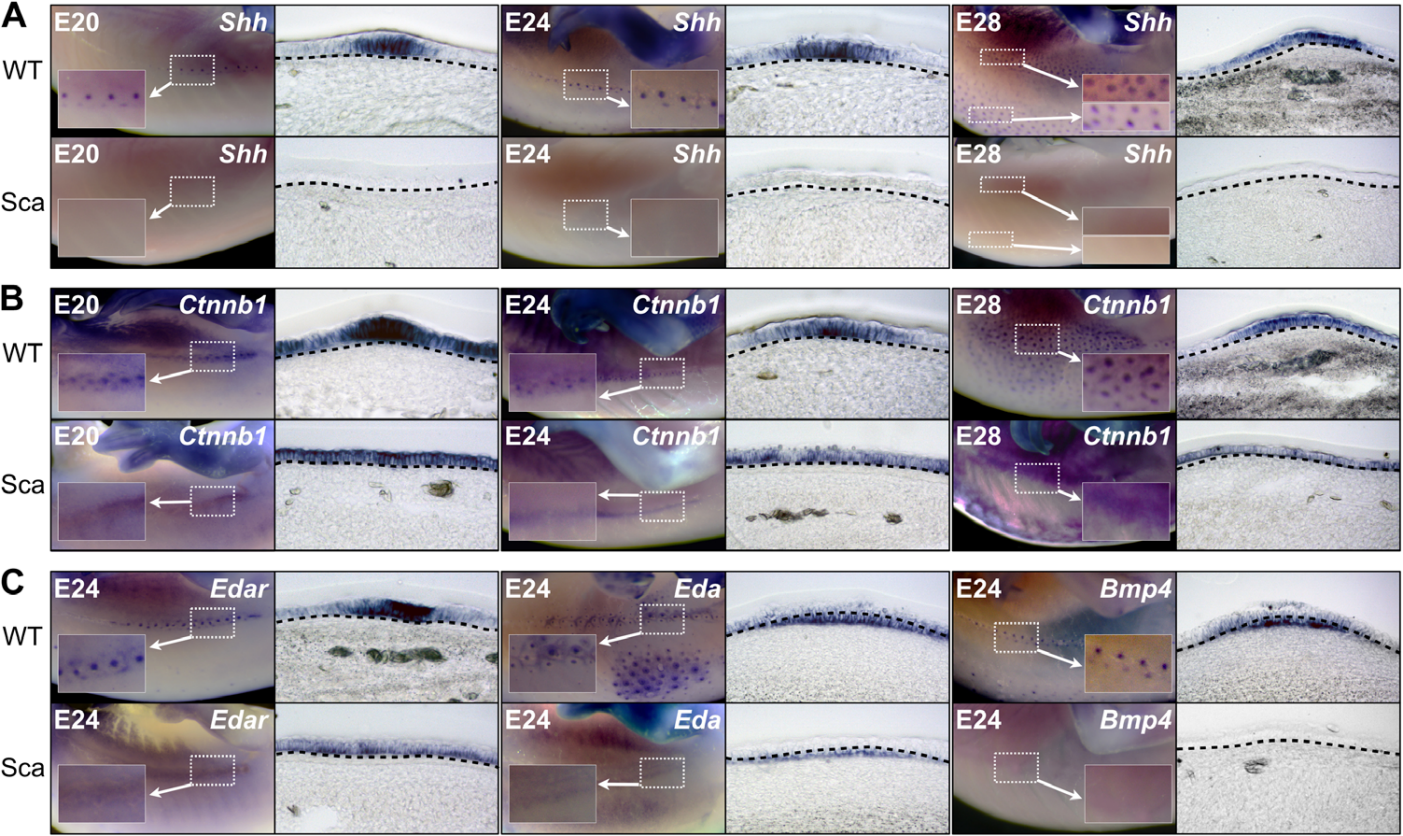
Absence of anatomical placodes in scaleless *P. vitticeps* skin. (**A** to **C**) WMISH showing the expression of early markers of epidermal appendage development in WT and Sca bearded dragon embryos at various indicated developmental stages: (A) *Shh*; (B) *Ctnnb1*; and (C) *Edar* (left), *Eda* (center), and *Bmp4* (right). Left panels show the WMISH signal on the lateral skin region, and right panels show parasagittal cryosections of the corresponding regions. Insets show high magnifications of the staining and indicate the presence/absence of placode formation in WT and mutant skin, respectively.

Scaleless dragons do no exhibit any *Shh* expression in the skin (Fig. 4A, lower panels), whereas the *Shh* expression dynamic in wild-type dragons is first restricted at the center of the placode before it spreads in a larger and more posterior domain (Fig. 4A, upper panels). Similarly, *Ctnnb1* and *Edar*, two other placode markers, also show marked differences in expression between wild-type and scaleless dragons. In both phenotypes, expression of these two genes is first ubiquitous across the whole epidermis before becoming restricted to the placodes in wild-type individuals only (Fig. 4B). These results indicate that expression of each of these three placode markers in reptiles is similar to the expression dynamic of the corresponding genes in mammals (*27*) and birds (*20*, *28*, *39*). On the other hand, the absence of an anatomical placode in scaleless dragons coincides with the inability of signaling pathways to pattern the skin, similar to what is observed in mice deficient in *Eda*/*Edar* (*40*). Note that both the functional Eda in wild-type dragons and the dysfunctional Eda in scaleless dragons are both expressed in the dermis [as in birds (*31*)], but the former remains diffused in scaleless lizards (Fig. 4C).

Finally, similar in situ hybridization analyses indicate the presence and total absence of *Bmp4* dermal expression in wild-type and scaleless dragons, respectively (Fig. 4C); this expression in wild-type dragons is additionally restricted in the dermis underlying placodes. These results indicate that all three characteristics (epidermal thickening, expression of epidermal placode markers, and expression of dermal Bmp4), that is, the presence of an anatomical placode, are required for proper development of scales in reptiles.

## DISCUSSION

The fossil record lacks any evidence of intermediate forms (hence, of homology) between scales and hairs. In addition, hairs in mammals, feathers and feet scales in birds, and scales in reptiles exhibit substantial differences in morphogenesis. Finally, the presence and absence of β-proteins [a family of proteins unrelated to α-keratins (*41*, *42*)] in skin appendages of sauropsids (birds and reptiles) and in those of synapsids (mammals), respectively, only added to the confusion. All these considerations have, for decades, fostered the debate on the homology, or lack thereof, among these skin appendages and led some authors (*3*, *5*, *8*) to conclude that homologous skin appendages do not exist beyond amniote classes (reptiles, mammals, and birds); that is, mammalian hair and avian feather would not have evolved from reptilian overlapping scales.

Several scenarios have been proposed to account for this hypothetical lack of homology. One model suggests (*3*, *5*) that mammalian hair evolved as mechanosensory appendages in interscale regions of the skin (that is, hinges of scales) of their reptilian ancestor and that more derived representatives of mammals lost their scales entirely while retaining their bristles that then increased in density and acquired their insulatory function. The second scenario (*8*) advocates that hair evolved from the glandular structure of stem amniotes, whereas early sauropsids lost these glandular structures and evolved a granulated β-keratinized integument, an innovation that would have allowed the independent evolution of squamate scales, crocodilian scutes, and avian feathers.

Both models assume that the development of an anatomical placode and of a dermal papilla occurred, at a minimum, twice (once in birds and once in mammals) through the independent parallel co-option of the same set of signaling pathways (WNTs, β-catenin, EDAR, BMPs, and SHH). Further fine-tuning of the expression levels of members of these pathways would then explain the diversification of skin appendage forms within lineages, such as the increase of β-catenin, causing the transition from feathers to avian scales (*19*) or from sebocytes to hair (*43*). Conversely, other authors argue that the similarities in molecular signaling observed among all skin appendages suffice to support their homology (*9*). Note that such placodal signaling centers have been recently evidenced as underlying the development of *Chelonia* shell scutes (*23*), although further analyses regarding the development of turtle scales elsewhere on the body are warranted.

The data presented here put this debate to rest by demonstrating that most skin appendages in amniotes, including snake and lizard overlapping scales, not only share signaling pathways during morphogenesis but also truly develop from anatomical placodes. The squamate anatomical placode, whose existence had remained undetected because of its transitory developmental dynamic, exhibits all the major features characterizing avian and mammalian placodes: local epidermal thickening with columnar cells and reduced proliferation rate, shared early spatially restricted expression of epidermal molecular markers, and localized conserved signaling in the underlying dermis. This latter point indicates that proper development of the reptilian scale placode requires, as for the avian and mammalian placodes, signaling interactions between the dermis and epidermis. Inactivation of the EDA pathway in the bearded dragon scaleless mutant disrupts these interactions, precluding scale morphogenesis.

In addition, the shared localized dermal signaling during the development of placodes in all amniotes makes the independent evolution of a dermal condensate in avian and mammalian follicles much less surprising than previously anticipated. The development of dermal osteoderms (*44*), which are associated with some epidermal scales in crocodiles and in some lizards, might even suggest that the dermal condensation abilities of the dermis constitute a deep homology among all amniotes. This hypothesis could be further tested by investigating, during reptile scale morphogenesis, the potential expression of other signaling molecules known to be dermal condensation markers in mammals and/or birds (*12*, *45*, *46*).

It has been previously hypothesized (*47*) that reptilian scales are more similar to avian reticulate scales (covering the foot pad) than to both avian scutate scales (covering the anterior metatarsal region) and feathers. Our results argue against that hypothesis as, contrary to avian reticulate scales, squamate scales, avian scutate scales, and avian feathers all form from an anatomical placode and all exhibit dermal signaling. Our results are consistent with the observation that reticulate scales are non-overlapping and composed only of α-keratin, whereas avian scutate and reptilian scales are mostly overlapping and composed of both α-keratins and β-proteins. Note that previous studies in chicken (including the mutant scaleless chicken) have shown that reticulate scales exhibit peculiar morphogenesis with alteration of proliferation patterns and of conserved signaling pathways (*8*, *25*, *48*), further suggesting that they are derived structures with little developmental similarities to reptilian scales.

## MATERIALS AND METHODS

### Animals

Fertilized eggs of Nile crocodiles (*C. niloticus*), corn snakes (*P. guttatus*), and bearded dragons (*P. vitticeps*) were incubated on a moistened vermiculite substrate at 29.5°C. Embryos were removed at different embryonic days after oviposition and were staged on the basis of their external morphology according to developmental tables available for crocodilians, lizards, and snakes (*49*–*51*). Maintenance of and experiments on reptilians were approved by the Geneva Canton ethical regulation authority (authorization GE/82/14) and performed according to Swiss law.

### Whole-mount in situ hybridization

Embryos at different developmental stages were fixed, and WMISH was performed as previously described (*24*). Species-specific digoxigenin-labeled antisense riboprobes correspond to Nile crocodile *Shh* [786 bp; coding sequence (CDS) region], Nile crocodile *Ctnnb1* [623 bp, 3′ untranslated region (UTR)], corn snake *Shh* (843 bp; CDS region), corn snake *Ctnnb1* (716 bp; CDS/3′UTR region), bearded dragon *Shh* (931 bp; CDS region), bearded dragon *Ctnnb1* (872 bp; CDS/3′UTR region), bearded dragon *Bmp4* (670 bp; CDS region), bearded dragon *Eda* (913 bp; CDS region), or bearded dragon *Edar* (1005 bp; CDS region). Corresponding sense riboprobes were used as negative controls. After WMISH, embryos were fixed in 4% paraformaldehyde, cryoprotected in 30% sucrose, embedded in optimal cutting temperature compound, and cryosectioned at 15 μm.

### Histology and immunofluorescence

Embryonic skin tissues from different body locations were fixed, sectioned (at 8 μm), and stained (with H&E or TB) as previously described (*24*). Immunofluorescence staining on skin paraffin sections was carried out as previously described (*24*), with one of the following primary antibodies known to recognize reptile and/or chicken epitopes: anti-PCNA (1:300; AbD Serotec), anti–pan-α-cytokeratin (1:50; Thermo Scientific), or anti-laminin Ab1 (1:100; Thermo Scientific). Last, incubation with the Alexa Fluor–conjugated secondary antibody (Alexa Fluor 488 or 568; Life Technologies) was performed for 1 hour. β-Proteins were detected by autofluorescence of untreated epidermis. Samples were mounted with DAPI (4′,6′-diamidino-2-phenylindole)–containing VECTASHIELD mounting medium (Vector Laboratories).

### Scanning electron microscopy and microcomputed tomography

For scanning electron microscopy, skin molts were mounted onto aluminum stubs with a conductive paste (carbon dag) and coated with gold using a Sputter Coater (JFC-1200, JEOL). Specimens were viewed and photographed using a JEOL 6510LV scanning electron microscope at an acceleration voltage of 10 kV. Micro–computed tomography scans of the cranial skeleton of newborn bearded dragons were performed using a SkyScan076 scanner with a pixel size of 18 μm, and three-dimensional reconstructions of the scans were generated using the Imaris software (Bitplane).

### Sequence analysis and semiquantitative RT-PCR

Genomic DNA and total RNA from embryonic wild-type and scaleless bearded dragon tissues were isolated using the DNeasy and RNeasy kits (Qiagen), respectively, according to the manufacturer’s instructions. cDNA was generated by reverse transcription using 2.5 μM oligo(dT) primer and 1 μg of total RNA (SuperScript kit, Invitrogen). The full-length cDNA of wild-type and scaleless *Eda* was isolated by PCR using sequences from the bearded dragon transcriptome (*52*) and by the subsequent rapid amplification of DNA ends (RACE kit, Roche). Intronic regions were obtained with PCR from genomic DNA using cDNA as a reference sequence. Exon boundaries were obtained by comparing the cDNA and genomic sequences. Semiquantitative PCRs on genomic DNA or cDNA were performed with the FastStart PCR system (Roche).
